# Adaptive Skills in FXS: A Review of the Literature and Evaluation of the PEDI-Computer Adaptive Test (PEDI-CAT) to Measure Adaptive Skills

**DOI:** 10.3390/brainsci10060351

**Published:** 2020-06-06

**Authors:** Lisa Cordeiro, Adrienne Villagomez, Deanna Swain, Sophia Deklotz, Nicole Tartaglia

**Affiliations:** 1 Department of Pediatrics, School of Medicine, University of Colorado, Aurora, CO 80045, USA; lisa.cordeiro@cuanschutz.edu (L.C.); Adrienne.Villagomez@childrenscolorado.org (A.V.); Sophia.Deklotz@childrenscolorado.org (S.D.); 2 Developmental Pediatrics, Children’s Hospital Colorado, Aurora, CO 80045, USA; dms4001@med.cornell.edu; 3 Department of Psychiatry, Weill Cornell Medicine, White Plains, NY 10605, USA

**Keywords:** adaptive skills, adaptive behavior, fragile X syndrome, PEDI-CAT, Vineland, clinical trials, reliability, feasibility, outcome measure

## Abstract

As adaptive skills (AS) are dynamic and may indicate the success of an intervention, they are a common domain measured in clinical trials. Typical interview tools for measuring AS are time-consuming, and questionnaire measures often lead to inconsistent information. The present study was designed to evaluate the feasibility, validity and test-retest performance of the Pediatric Evaluation of Disability Inventory Computer Adaptive Test (PEDI-CAT) in Fragile X syndrome (FXS). The PEDI-CAT is administered via tablet and uses the item response theory to efficiently determine the items administered. The PEDI-CAT was administered to 42 individuals with FXS (27 males; 15 females) aged 1.6–50.9 years (M = 14.9; SD = 11.2), followed by the Vineland-3 (VABS-3) interview for comparison. Administration was efficient (M = 21.7 min; SD = 9.5; range 8–45 min; mode = 19). Males and females did not significantly differ on the PEDI-CAT domains, except for daily activities (t(40) = −2.22, *p* = 0.037). Floor effects were significant for both measures, although the PEDI-CAT showed more floor effects in the mobility (35.7%) and social-cognitive (50%) domains. PEDI-CAT daily activities, mobility, social-cognitive and responsibility domains were all significantly correlated with most of the VABS-3 domains (all rho > 0.5; *p* < 0.01). Test-rest of the PEDI-CAT was comparable to the VABS-3. Results suggest that the PEDI-CAT is efficient, and minimal training is needed to administer it; however, it lacks specificity and shares a high rate of floor effects with the VABS-3.

## 1. Introduction

Fragile X syndrome (FXS) is the most common known inherited cause of intellectual disability (ID) and autism spectrum disorder with an estimated frequency of about 1:4000–5000 [1] affecting all racial and ethnic groups worldwide. Extensive study of the neurobiology and synaptic mechanisms of the disease in cellular and animal models has identified many neuronal targets for treatment and made possible enormous progress in preclinical and clinical translational works in FXS over the past decade [2,3]. Early clinical trials of several new treatments targeted to the underlying disease have been initiated, and some new drugs targeting glutamate or GABA pathways have shown promising results in both open-label and early phase studies [4,5]. Multiple complex issues have led to challenges replicating results in larger placebo-controlled phase 2b and 3 trials [6,7], including the lack of outcome measures validated in FXS [8]. Further, most previous trials have focused on behavioral outcomes in short length studies; however, the FDA has emphasized the importance of trials evaluating changes in outcomes that reflect changes to functional outcomes and quality of life [9]. Adaptive functioning measures may also better reflect the overall functioning of individuals with FXS compared to cognitive measures, given challenges with the administration and interpretation of standardized cognitive tests in much of the FXS population, as well as the effects of anxiety and avoidance on test performances [10]. As a result, the validation of measures of adaptive functioning for use in clinical trials is critical to show whether intervention leads to clinically meaningful changes in day-to-day functioning.

In FXS, the Vineland Adaptive Behavior Scales is consistently utilized in large-scale national research efforts [8,9] and largely considered a gold standard measure of adaptive behavior [11,12]. However, the Vineland can be lengthy to administer by interview, ranging between 30 and 90 min, depending on age. This time requirement increases the burden on research participants and increases research costs. While the Vineland is also available as a parent/caregiver report survey, the instructions are not clear to many parents, and results are often inconsistent compared to those obtained through interview. Given these obstacles, the purpose of the present study is to examine the feasibility, validity and reliability of a computerized adaptive measure of adaptive functioning in FXS as a potential outcomes measure for both clinical care and research trials.

### 1.1. Pediatric Evaluation of the Disability Inventory-Computer Adaptive Test (PEDI-CAT)

The PEDI-CAT, a revised and updated version of a parent- or clinical-report measure called the PEDI [13], measures function and participation across four domains (i.e., daily activities, mobility, social-cognitive and responsibility) in individuals from birth to 21 years of age [14]. Within each domain, there are subdomains called content areas that explore different skills falling within that domain. Currently, there are two versions of the PEDI-CAT (i.e., speedy and content-balanced or comprehensive). Compared to 15 items per domain for the speedy version, the content-balanced version uses approximately 30 items per domain, which provides questions from each content area within each domain (e.g., items from each of the four content areas within the daily activities domain: (1) getting dressed, (2) keeping clean, (3) home tasks and (4) eating and mealtime).

The initial item pools for the PEDI-CAT were developed through a comprehensive review of over thirty existing measures, such as the Vineland and Scales of Independent Behavior (SIB-R) [14]. Items are worded using everyday language, and clear examples and illustrations of items are included on the device screen to facilitate understanding of the item’s intent. It was normed with a large sample of neurotypical children (*n* = 2205) and children with disabilities (*n* = 703) and demonstrated excellent accuracy (intraclass correlation coefficients (ICCs) ≥ 0.95) with the full item banks [15,16]. Other research teams have reviewed items in the PEDI-CAT manual and found strong evidence for convergence with the activity and participation components of the International Classification of Functioning, Disability and Health [17,18].

The PEDI-CAT has been translated and cross-culturally adapted into several versions, including Spanish, Dutch and Brazilian-Portuguese [19,20]. It has been used to assess motor functioning in children with learning disorders and children with spina bifida, as well as all domains of functioning in autism spectrum disorder (ASD), Down syndrome, cerebral palsy, Angelman syndrome, infants and toddlers receiving EI services and many others [21,22,23,24,25,26,27,28,29,30,31,32]. Several studies show strong psychometric evidence for the PEDI-CAT. For example, researchers have shown good construct (convergent and divergent) validity for the PEDI-CAT (speedy version) in children with high and low levels of medical complexity, as well as cerebral palsy [28,33,34]. However, other studies show mixed findings when examining the content validity of the PEDI-CAT within specific populations and concurrent validity (i.e., comparisons with “gold standard” measures assessing similar constructs). For example, Dumas and colleagues [35] compared the performance of the PEDI-CAT mobility domain (speedy version) to the Alberta Infant Motor Scale (AIMS; [36]) in 53 infants and toddlers. Results showed a significant but weak association between measures. Both instruments demonstrated a responsiveness to change over time. The AIMS, however, identified more children as delayed compared to the PEDI-CAT. Additionally, when exploring the content validity of the mobility domain (speedy version), several researchers identified a floor effect, indicating items may be needed on the lower end of the measure [37].

The PEDI-CAT uses item response theory (IRT) statistical models. Measures that use IRT are designed to provide an accurate and precise assessment while increasing the efficiency and reducing the respondent burden. Additionally, computerized adaptive testing (CAT) applications provide a unique feature that allows for additional specialized items to be added for target populations. In 2015, the PEDI-CAT was adapted for the ASD population by including additional question items and directions [38]. Kramer and colleagues [25] examined the test-retest and concurrent validity of the new version (PEDI-CAT-ASD), a speedy version in 39 children and adolescents with autism spectrum disorder (ASD). Results demonstrated a reliability for all PEDI-CAT-ASD domain scores (ICC > 0.86) and a high correlation to VABS-2 domain raw scores. However, when assessing the functional abilities of preschoolers referred for diagnosis due to pediatrician concern, the PEDI-CAT (speedy version) was less sensitive in identifying those in need of support compared to the Vineland-3 [39]. Due to discrepant findings across studies, a specific study of the PEDI-CAT in the FXS population compared to well-validated measures is important.

### 1.2. Review of Previous Research on Adaptive Behaviors in FXS

Extensive research in FXS has identified profiles of adaptive behaviors, patterns of strengths and weaknesses (for review, see Hahn et al., 2015 [40]) and developmental trajectories within FXS [15,16,40,41,42]. Though findings are mixed, daily living skills are generally considered an area of relative strength, with lower scores in communication and social skills for individuals with FXS [15,41,42,43,44,45,46]. When the FXS sample is differentiated by co-occurring ASD (FXS + ASD), research suggests different patterns of strengths and weaknesses. While young children aged 21 to 48 months old with FXS exhibited an even developmental profile on the Vineland, children of the same age with FXS + ASD showed strengths in motor and daily living skills relative to their social and communication skills [46]. Similarly, as children age, those with FXS + ASD continue to show motor skills as an area of relative strength, with more significant difficulty in social and communication skills [15]. Those without co-occurring ASD show higher scores and rates of growth on daily living skills, yet also with the lowest scores in socialization [15]. In others studies that did not distinguish between FXS and FXS + ASD, socialization skills on the Vineland were identified as a strength relative to daily living skills and communication skills [16,42].

Adaptive behavior outcomes in FXS are impacted by several variables, including gender and age. As would be expected, females typically score higher across adaptive behavior domains [15]. Age as a factor related to adaptive behavior is best understood by examining developmental trajectories in FXS [42]. While many studies suggest general declines in adaptive behavior over time [16,41,47,48], findings are mixed. Differences in developmental trajectories are often attributed to factors including gender; age; number of time points that skills were measured and type of scores used (i.e., standard scores, age equivalents and raw scores) [40]. Both males and females with FXS show a skill acquisition rate that slows over time [15,16,47,49]. Steady increases of adaptive behaviors for individuals with FXS are often seen until 10 to 12 years of age before skills plateau or decline, regardless of the type of scores analyzed [15,40,41,44,50]. For children who showed raw score declines in adaptive behaviors, many showed declines in socialization [40]. In one longitudinal study with individuals followed through 18 years of age, males with FXS shows significant declines in their standardized scores across all adaptive behavior domains and females with FXS showed significant declines in communication [16]. It is important to note that, while research generally indicates declines in adaptive behaviors over time, some studies suggest that adolescents and adults continue to develop functional, daily living skills across their lifespans [16,51]. One survey of families of adults with FXS found that functional (daily living) skills were the strongest predictor of independence in males, whereas interpersonal skills were found to be the strongest predictor of independent living for women [52]. The majority (over 85%) of adults with FXS aged 20 years and older had mastered many core daily living skills, including eating, dressing, toileting and bathing [51]. As expected, however, while the majority of adult females attained complex functional skills in communication and self-care, 60% or fewer adult males mastered similar skills [51]. Less than half (43.8%) of women with FXS and nearly one in ten (9.1%) men with FXS achieved “high” or “very high” levels of independence in their adult lives [52].

Other features of the FXS behavioral phenotype, including intellectual disability, ASD, social anxiety and problem behaviors, also contribute to variability in adaptive behaviors [10,15,47,51,53,54,55,56]. Of these, cognitive ability and ASD are considered the strongest predictors. There is a strong and complex relationship between a cognitive ability and adaptive behavior, with some research suggesting that adaptive behaviors may surpass nonverbal cognitive abilities in FXS [15,41,57]. In young children with FXS, nonverbal cognitive abilities impact the rate of growth in adaptive behaviors over time [40]. Similarly, in females with FXS, there is a strong, positive relationship between nonverbal abilities and adaptive behaviors [58]. Interestingly, however, relative cognitive strengths in verbal abilities in FXS [59,60,61,62] do not appear to translate to outcomes in adaptive behaviors [58]. In addition to intellectual disability, the degree of autism symptoms in individuals with FXS significantly and negatively impacts independent living skills for both males and females [15,63]. Moreover, this finding is “syndrome-specific”; when compared to age-matched individuals with ASD without FXS, the ASD symptom severity had a stronger influence on independent living skills for individuals with FXS [63]. Taken together, children with FXS with higher nonverbal cognitive abilities and fewer ASD symptoms tend to show higher adaptive behavior trajectories than children who present with more difficulties [15,40,44,46,50,61]. In addition to ASD, social skills and symptoms of anxiety and depression have been found to be negatively correlated with adaptive behaviors for females with FXS [55,58,64]. Increasingly, research has also explored environmental variables that are predictive of adaptive behaviors in FXS, including the quality of the home environment and parent responsivity [49,65]. In comparison, the caregiver education level was not associated with functional skills for individuals with FXS across their lifespans [51].

### 1.3. Present Study

The purpose of this study was to explore the results of a computer-adaptive measure of adaptive behaviors (PEDI-CAT) [14] in individuals with FXS compared to the Vineland-3.

### 1.4. Research Questions

1. How feasible is it to utilize the PEDI-CAT in a research trial?
2. Is the PEDI-CAT a valid measure of adaptive behaviors when compared to the gold standard (Vineland-3) for individuals with FXS?
3. What is the test-retest reliability of the PEDI-CAT?

## 2. Materials and Methods

### 2.1. Study Design

#### 2.1.1. Recruitment

Participants were invited to participate in an IRB-approved study evaluating outcome measures and on the longitudinal phenotypes of FXS across the lifespan. The study was carried out at the University of Colorado and Children’s Hospital of Colorado (CHCO) Denver Fragile X Clinic. Inclusion criteria for the study included males and females with FXS thirty-one days to sixty-five years old who were already enrolled or agreed to enroll in the national Fragile X Online Registry with Accessible Research Database (FORWARD) project. FORWARD began in 2012 and consists of a patient and family registry plus a longitudinal database, which includes clinician- and parent-reported data about individuals living with FXS at over 25 sites across the US [66]. Both FORWARD and Component C (grant number 1U01DD001190) are funded by the Centers for Disease Control and Prevention (CDC). Participants were recruited from the Denver Fragile X Clinic and via multiple national advocacy and support organizations for FXS. All participants or their parents signed an IRB-approved consent form prior to participation. Medical records and genetic testing results were reviewed to confirm a diagnosis of FXS. For this project, results from two years of the study were included in the analysis.

#### 2.1.2. Evaluation

A comprehensive core battery of assessments was administered to participants on an annual basis, dependent on age, to characterize cognitive, language, behavioral and adaptive functioning skills.

##### Vineland Adaptive Behavior Scales-Third Edition, Comprehensive Interview (VABS-3)

The Vineland [11] is a comprehensive adaptive behavior measure that yields composite standard scores in the domains of: communication (receptive, expressive and written adaptive language functions); daily living skills (personal, domestic and community skills); socialization (interpersonal relationships, play and leisure time and coping abilities) and motor skills (gross and fine motor skills). All domains except the motor skills domain are included in an overall score, referred to as the adaptive behavior composite (ABC). The motor skills domain is optional [11,12] and normed for individuals 9 years and younger. The VABS-3 has excellent internal consistency on the comprehensive form, with coefficients ranging from 0.94 to 0.99 and test-retest reliability ranging from 0.64 to 0.94 [67]. The VABS-3 concurrent validity between domains ranged from 0.32 to 0.83 [67]. The VABS-3 interview forms were administered by in-person interview with parents or caregivers during research visits after administration of the PEDI-CAT. Average administration time for the VABS-3 interview depends on the age of the person being evaluated and can range from 30–45 min for very young children to 60–75 min for older children, adolescents or adults.

##### The Pediatric Evaluation of Disability Inventory-Computer Adaptive Test (PEDI-CAT)

The PEDI-CAT consists of four domains: daily activities, social-cognitive, mobility and responsibility. The daily activities domain is comprised of four content areas, including getting dressed, keeping clean, home tasks and eating and mealtime. The social-cognitive domain includes content areas of interaction, communication, everyday cognition and self-management. The mobility domain includes five content areas, including basic movement and transfers, standing and walking, steps and inclines, running and playing, and wheelchair (as appropriate to each child). The responsibility domain measures the extent to which the caregiver or child takes responsibility for managing complex, multi-step life tasks in the areas of organization and planning, taking care of daily needs, health management and staying safe. In the three functional skill domains of daily activities, mobility and social-cognitive, a caregiver selects their response from a 4-point scale of difficulty, rating the child’s ability ranging from “unable” to “easy”. The responsibility domain uses a 5-point scale, with response choices rating how much responsibility both the child and caregiver each assumes. Responsibility domain responses range from “adult/caregiver has full responsibility; the child does not take any responsibility” to “child takes full responsibility without any direction, supervision or guidance from an adult/caregiver” [14]. The PEDI-CAT has high test-retest reliability (ICC = 0.96–0.99) for all four domains [14].

The content-balanced (“comprehensive”) PEDI-CAT consists of roughly 30 items per domain, including a balance of items from each of the content areas within each domain. The score report for the comprehensive PEDI-CAT yields the following: (1) scaled score that is not age-related and represents each child’s performance along the continuum of function. Differences in scaled scores represent the absolute amount of change that has occurred from one visit to another and are recommended to track functional progress. (2) T-score (M = 50; SD = 10; typical range= 30–70)) that describes a child’s performance in comparison to other children of the same age (by one-year intervals), and (3) age percentile category ( <5th, 5th–25th, 26th–50th, 51st–75th and >75th percentiles). A fit score for the scaled scores and standard error (SE) for the T-scores are also reported.

In the present study, the content-balanced (“comprehensive”) PEDI-CAT was administered, because it is used for individual program planning and may be relevant for monitoring changes within an individual in the context of a clinical trial. The PEDI-CAT was administered on a computer tablet that was provided to the parent or caregiver, and instructions were provided by the study coordinator. The PEDI-CAT was completed before administration of the Vineland interview, in order to evaluate if the PEDI-CAT captured valid adaptive functioning scores before caregivers were led through a structured Vineland interview reviewing and clarifying adaptive functioning skills. To get the purest assessment of the PEDI-CAT measure, independent of the Vineland, it was administered first. All aspects of PEDI-CAT administration (time to complete, understanding, consistency, response choices, etc.) could have been impacted if the Vineland had been administered on the same day and before the PEDI-CAT, particularly since the study used the interview format that requires the interviewer to probe for clarification about specific independent skills.

##### Stanford Binet Intelligence Scales, Fifth Edition (SB-5)

The SB-5 [68] is a standardized measure of cognitive functioning. The SB-5 provides scores for verbal and nonverbal abilities across five domains: fluid reasoning, knowledge, quantitative reasoning, visual-spatial reasoning and working memory, through which a full-scale IQ composite score is derived. The SB-5 has high reliability, internal consistency and well-documented criterion and concurrent validity. In this study, deviation scores were derived using an age-dependent (within each population age band) z-score transformation previously described [69] to generate a nonverbal IQ (NVIQ), verbal IQ (VIQ) and full-scale IQ (FSIQ) score for those participants who were administered the complete SB-5 test. As some participants completed only the routing subtests of the SB-5 due to an inability to complete all domains of the full SB-5, the abbreviated IQ (ABIQ) is also reported.

##### Bayley Scales of Infant and Toddler Development, Third Edition (BSID-3)

The BSID-3 [70] is a developmental assessment that measures cognitive, language (receptive and expressive) and motor (fine and gross) skills in children from birth to 42 months old. The BSID-3 has high internal consistency, test-retest reliability and validity [71,72]. Three female participants who were too young for the Stanford Binet were administered the BSID-3 for cognitive assessment.

##### Aberrant Behavior Checklist-Community Edition (ABC-C)

The Aberrant Behavior Checklist [73] is a caregiver rating scale developed initially to assess problematic behaviors in individuals with intellectual disabilities. The ABC-C includes five original subscales: irritability, lethargy, stereotypy, hyperactivity and inappropriate speech [73]. The ABC-C has excellent reliability and validity [73,74,75,76]. Previous studies in FXS suggest a good test-retest reliability of the ABC-C [77].

Modified subscales of the ABC-C for FXS were proposed to include six subscales: irritability, socially unresponsive/lethargy, hyperactivity, inappropriate speech and social avoidance based on a factor analysis [78]. In the modified ABC-C for FXS, several items of the hyperactivity subscale were shifted to the irritability subscale. The sixth subscale, social avoidance, includes four items taken from the original lethargy/withdrawal subscale [78]. Strong internal consistency for the modified factor structure including the six subscales has been demonstrated [79]. Several FXS studies have shown that the ABC-C distinguished males with FXS from males with FXS and co-occurring ASD [80,81,82]. Similarly, total problem behaviors on the ABC-C were strongly associated with autistic behaviors in males with FXS [83].

### 2.2. Analysis

All analyses were conducted with SPSS, version 26.0 (IBM, Armonk, NY, USA). Descriptive statistics of measures were calculated for males, females and the total sample. Independent sample two-tailed *t*-tests and Fisher’s exact tests were used with continuous and categorical variables, respectively, to examine differences between males and females and differences between VABS-3 and PEDI-CAT floor effects. PEDI-CAT total administration time was calculated using the start and stop times exported from the PEDI-CAT software program. Numerous analysis steps were carried out to assess the feasibility, validity and test-retest reliability, as described below. Separate analyses were conducted for those age-appropriate for the measures (ages < 21 years) and those outside of this age range (ages > 20 years, *N* = 8).

#### 2.2.1. Feasibility Analysis

Length of administration, training of research staff and use of the PEDI-CAT across the FXS phenotypes were used to determine the feasibility of the PEDI-CAT in this sample.

#### 2.2.2. Concurrent and Divergent Validity Analysis with VABS-3

Validity refers to the degree to which the evidence and theory support the interpretations of test scores for the proposed use of the test (i.e., the degree to which a test measures the construct(s) that it claims to measure). Convergent validity describes the extent to which different methods of measuring the same trait yield similar results. While divergent validity examines whether constructs that should have no relationship do, in fact, not have any relationship. We determined the convergent and divergent validity of the PEDI-CAT by comparing the PEDI-CAT scores with those of an established test of adaptive behaviors with known validity (VABS-3) using nonparametric correlations. Nonparametric correlations were used due to the uneven sample size and to ensure that outliers did not disproportionally influence the results.

#### 2.2.3. Test-Rest Reliability

The stability of the test scores over time was examined on a group of nineteen (*N* = 19) individuals who were administered the PEDI-CAT on two occasions (time 1 and time 2) 12 months apart within the ongoing longitudinal phenotyping study. The respondent was the same individual for both time 1 (test) and time 2 (retest) administrations. A two-way mixed effects single measurement absolute agreement model was used to calculate the ICCs.

## 3. Results

### 3.1. Participants

A total of forty-two participants (27 males) completed the PEDI-CAT, and most (*N* = 39) also completed the VABS-3. Missing data across all measures was primarily due to limitations on the length of assessments tolerated by participants with FXS, as well as the availability of measures (i.e., newest edition of the VABS) at the beginning of the study. Of the total sample, nineteen were administered retest PEDI-CAT, and twelve had both PEDI-CAT and VABS-3 retests available. Females had significantly higher IQ (ABIQ: t(35) = −3.44; *p* < 01) and VABS-3 scores (across all domains, see Table 1) and lower ABC total scores (t(25) = 2.348; *p* < 0.05). Three females completed the BSID-3 (cognitive: M (3) = 91.25 ± 12.5, R (75–105)) and two males did not complete the SB-5. Males and females did not have significantly different scores on the PEDI-CAT, with the exception of a significantly higher daily activities T-score among females (t (40) = −2.222; *p* < 0.05) (see Table 1 and Table 2). Eight participants over age twenty were administered the PEDI-CAT and scored according to the highest age band (20 years). This group had significantly lower PEDI-CAT mobility (t (40) = −2.254; *p* < 0.05), social-cognitive (t (40) = 2.170; *p* < 0.05) and responsibility T-scores (t(40) = 2.565; *p* < 0.01) compared to the standard age group.

### 3.2. Feasiblity of the PEDI-CAT in FXS

The PEDI-CAT was administered by research staff with a range of education and experience (research assistant to MD) after a short (~30 min) training session on how to set up the software and give instructions to the parents to complete the assessment. Administration time of the PEDI-CAT by parents ranged from 8–45 min (Mean = 21.4; SD = 9.5; mode = 19).

### 3.3. Validity of the PEDI-CAT in FXS

The correlation coefficients between each of the PEDI-CAT T-scores and VABS-3 domain standard scores are presented in Table 3. Numerous significant positive correlations exist between the two measures across all subdomains, showing convergent validity; however, also limited divergent validity. PEDI-CAT daily activities were significantly correlated with VABS-3 daily living skills (rho(13) = 0.664; *p* < 0.01), but this was not the strongest of the correlations with the VABS-3 (strongest relationship of PEDI-CAT daily living skills was with VABS-3 communication) and did not remain significant after control for multiple comparisons. The relationships between PEDI-CAT daily activities and VABS-3 communication, socialization, motor and ABC standard scores did remain significant after control for multiple comparisons. This supports poor divergent validity for the PEDI-CAT daily activities domain.

PEDI-CAT mobility had significant relationships of similar strength (rho R (0.511–0.551)) with VABS-3 adaptive composite (ABC), socialization and communication, which did not remain significant after control for multiple comparisons. The social-cognitive domain of the PEDI-CAT was most strongly correlated with VABS-3 communication (rho (13) = 0.711; *p* < 0.01) but was also significantly correlated with all of the VABS-3 standard scores. The relationship between social-cognitive and VABS-3 communication and ABC scores remained significant after control for multiple comparisons. PEDI-CAT responsibility T-score was significantly correlated with VABS-3 communication, socialization and ABC standard scores, with relationships of similar strength (R (0.618–0.678)), of which, communication remained significant after control for multiple comparisons. Responsibility was not significantly correlated with VABS-3 daily living skills or motor standard scores.

We also examined differences in floor effects between the PEDI-CAT and VABS-3, as shown in Table 3. For the PEDI-CAT, basal scores occurred for 26.2–50.0% of participants depending on the domain, compared to 10.0–35.9% across different VABS-3 domains. For the VABS-3, the percentage of individuals demonstrating a floor effect for the communication domain was 35.9%, with subscales ranging from 34.2% in receptive language to 50% in expressive language. For the daily living skills domain, 24.3% received basal scores, ranging from 19.4% in domestic to 31.6% in personal. Within the social domain, 16.2% of participants were identified as receiving the lowest scores, ranging from 11.1% in coping to 32.4% in play and leisure. Finally, in the motor domain, 10% of participants showed a floor effect, ranging from 13.3% in gross motor to 26.7% in fine motor. When directly comparing similar domains from the PEDI-CAT and VABS-3, significantly more participants earned floored scores on the PEDI-CAT mobility domain compared to the VABS-3 motor skills domain (*X^2^* (1, *N* = 72) = 6.17; *p* < 0.01), as well as on the PEDI-CAT social-cognitive domain compared to the VABS-3 socialization domain (*X^2^* (1, *N* = 79) = 9.98; *p* < 0.01). The distribution of participants earning the lowest score on other similar domain comparisons were not significantly different on the PEDI-CAT versus the VABS-3 (see Table 4). Overall, more participants earned floored scores on the PEDI-CAT motor and social-cognitive domains compared to similar domains on the VABS-3; however, the high percentage of floor effects for both measures are important to note.

### 3.4. Test-Retest Reliability of PEDI-CAT in FXS

Both the correlation and the intraclass correlation coefficient (ICC) between time 1 (test) and time 2 (retest) scores on the PEDI-CAT were strong across all four domains (daily activities, mobility, social-cognition and responsibility). Correlations between time 1 and time 2 scores ranged from 0.644–0.957, with the mobility score having the weakest relationship. All correlations remained significant after control for multiple comparisons (PEDI-CAT *p* < 0.006 and VABS-3 *p* < 0.01).

The PEDI-CAT stability coefficients at the domain level ranged from ICCs of 0.665 to 0.929, with an average ICC across all domains = 0.841. Test-retest reliability for the mobility score was poor (ICC = 0.665), while ICCs for all other scores were good (ICC = 0.75–0.90) to excellent (ICC ≥ 0.90). PEDI-CAT test-retest reliability was comparable to ICCs of the VABS-3 domain standard scores (ICC = 0.769–0.910). These results are shown in Table 5.

## 4. Discussion

### 4.1. Summary

Overall, this project evaluated adaptive skills in FXS using the PEDI-CAT in comparison to the VABS-3 as the gold standard to determine strengths and weaknesses of the measure in FXS and for consideration as a possible outcome measure for use in research studies. We first compared overall results and score profiles compared to previous literature. In this sample, adaptive score profiles on the VABS-3 are comparable to previous studies that have identified daily living skills and motor skills as areas of relative strength and communication as an area of weakness in both males and females [15,41,42]. The VABS-3 profile in our sample differs from most previous studies in that the mean socialization score was also a relative area of strength rather than an area of weakness, although this strength has been found in other, smaller FX samples [15,43]. VABS-3 scores also compared to previous studies showing females with FXS having significantly higher scores in all adaptive domains compared to males [43,44,45,46]. In comparison, the PEDI-CAT also identified strengths in the corresponding domains of mobility, responsibility (which includes many daily living skills) and daily activities, with social-cognitive (which contains communication skills) as the lowest domain. While there were trends toward lower scores in males in the PEDI-CAT domains compared to females, in this sample, they only reached significance in the daily activities domain. While these differences may be more pronounced on the PEDI-CAT in larger sample sizes, these findings suggest that the PEDI-CAT may not be as sensitive to the previously reported gender differences of adaptive behaviors in FXS, or differences among content areas across the two measures may impact comparisons of adaptive skill profiles by gender.

The PEDI-CAT administration was more efficient than the VABS-3 interview and was a major advantage, with minimal training needed for the research staff to administer compared to the VABS-3 and, also, only taking approximately 20 min for the caregiver compared to 30–60+ min for the VABS-3, requiring both staff conducting the interview and the caregiver. This difference can be significant when considering many factors, including that a child with FXS would then often need another adult for supervision while the VABS-3 was conducted, and the longer duration of time would extend the time of the research or visit. Fatigue on the part of both the participant and caregiver can then decrease the ability to tolerate or perform optimally on other assessment measures or procedures and, also, lead to negative experiences with the visit, which could subsequently affect research retention and discourage future participation. While these measures were administered as part of a research battery, the same considerations related to time and burden can be consideration in the clinical setting, where measurements of adaptive functioning are a frequent and important component of FXS assessments.

For the small subgroup over age twenty years old, the highest age band used for scoring did not result in similar PEDI-CAT score profiles as those under age twenty-one, and this difference was not explained by lower IQs in the older age group. The significantly lower PEDI-CAT scores in this older age group indicates that it is not appropriate to administer beyond the normative age range without the establishment of norms for an older age group.

When we compared the PEDI-CAT to the VABS-3 scores, there were many significant correlations between the PEDI-CAT T-scores and VABS-3 standard scores across multiple domains and subdomains (Table 3). These results signal strong concurrent validity but, also, limited divergent validity between the two measures. For example, we hypothesized that daily activities from the PEDI-CAT would be most strongly correlated with the daily living skills domain on the VABS-3. Although a significant relationship between the PEDI-CAT daily activities T-score and VABS-3 daily living skills standard score was found, this did not hold up after control for multiple comparisons. Further, daily activities were more strongly correlated with the other VABS-3 domain standard scores, and these relationships did endure after correction.

In a study comparing the VABS-2 and PEDI-CAT (speedy version developed for ASD) in a group of children with autism (just over 50% of our sample had an autism diagnosis), it was posited that differences in content structures between the PEDI-CAT and VABS may explain the limited divergent validity and high levels of correlation across domains that were also found in our study [24]. Consistent with our results, the study in autism reported the PEDI-CAT social-cognitive domain to be significantly correlated with all of the VABS domains. Kramer suggested that this may due to the indirect inclusion of interpersonal and communication skills on many VABS items, even those from other domains [24]. The Kramer study reported expected convergent and divergent validity between PEDI-CAT daily activities and VABS-2 domains, which were not replicated in our study. This may be due to the additional word changes on the PEDI-CAT ASD version used in the Kramer study that were made by the publishers to focus on performance and remove communication and interactions from item descriptions so they were more aligned with the ASD population. This warrants future investigation of this PEDI-CAT ASD version in FXS.

The PEDI-CAT was designed to be consistent with the World Health Organization’s International Classification of Functioning, Disability and Health for children and youth (ICF-CY) [14,18]. Briefly, the ICF-CY was developed to provide a universal framework for disability and functioning. While VABS-2 concepts are generally represented in the ICF-CY structure, about 30% of VABS-2 concepts do not align [84]. As ICF-CY emphasizes successful outcomes of performance and discrete skills (not method), the VABS-2 item requirements imply specific methods of performance and/or include multiple concepts in one item. The revision of the VABS to the third edition included adjustments that allow more flexibility in the method (i.e., use of a communication device), which may better align with ICF-CY concepts. However, the VABS-3 includes concepts that represent some of the core impairments in autism (i.e., theory of mind, social awareness, attachment, etc.), and these are not included in ICF-CY concepts. This may impact the comparability of the two measures generally, as well as in our study sample.

As floor effects are a recurrent issue in the assessment of individuals with an intellectual disability, we compared how many participants earned the lowest score on the PEDI-CAT (T-score ≤ 10) or the lowest possible standard score on the VABS-3 (standard score = 20). Both T-scores on the PEDI-CAT and standard scores on the VABS-3 domains are based on comparisons to a normative population, and individuals with ID may score below what is measurable on either test. We found that more participants floored across the PEDI-CAT domains compared to the VABS-3, especially when comparing the social-cognitive (47.4%) and socialization (13.5%) domains. However, overall, both measures showed marked floor effects, with about one-third of the sample showing floor effects on both measures in at least one subdomain. Further, while two of the five domain comparisons revealed that significantly more participants scored at the floor on the PEDI-CAT, the remaining three domain comparisons were not significantly different between the PEDI-CAT and VABS-3. Other studies have also found that there are elevated floor effects using the PEDI-CAT in populations with significant functional needs [31,39] and likely represents insufficient items at the lower end of the assessments. These results highlight the need for additional measures or the validation of new scoring methods for both of these measures if adaptive domains are included in a research trial, as these measures would not likely capture changes well for this lower functioning subset of patients with the current scoring systems.

There are clear differences in the content and conceptual frameworks of the VABS and PEDI-CAT, as well as the methods of administration and scoring. Whereas the VABS asks the caregiver to rate the frequency that their child does an activity independently, the PEDI-CAT asks the caregiver to evaluate the level of difficulty that the participant encounters during an activity. As a computer adaptive measure using the item response theory (IRT), the PEDI-CAT content-balanced version (used in the present study) displays items in an order dependent upon the response choice selected on the previous item, as opposed to a consistent order on the VABS-3.

The test-retest reliability (by both correlations and ICCs) was strong for the PEDI-CAT and VABS-3 in our sample, with an interval of 12 months between measurements. Among the PEDI-CAT domains, the mobility scaled score (similar to a weighted raw score) had the weakest test-retest reliability. Many clinical trials are a much shorter duration, however, and often track raw score changes rather than standard scores. Further research would be needed for evaluating the PEDI-CAT performance in trials of shorter durations before consideration of inclusion in a clinical trial. An important difference of the PEDI-CAT is that items are selected by IRT algorithms, and, thus, not all items are consistent between administrations. For an interval of 12 months between measurements, this did not lead to a marked variability in scores; however, in a trial of a shorter duration, more variability could be introduced if the same items were not asked at subsequent administrations. Overall, in FXS where behavior and skills can be so variable, and considering the importance of consistency in clinical trial data collections, investigators selecting an adaptive measure should evaluate the individual items and the IRT algorithms (if present) to make sure they are appropriate for the FXS study sample and research question of their study.

### 4.2. Limitations

While our overall sample size was of adequate power for the calculation of the test-rest reliability, an increased sample of females, additional participants with VABS-3 scores at time 2 and a broader representation of age groups would have allowed for additional analyses and stronger conclusions regarding the performance of the PEDI-CAT across the FXS phenotype. These analyses will be possible with ongoing data collection in this longitudinal study.

Overall, a limitation of both the PEDI-CAT and VABS-3 (and other standardized measures of adaptive functioning) is that the results are dependent on parent reports rather than direct observations of skills. While parents are the most likely reporter with knowledge of their child’s skills across the domains and the concept of adaptive functioning includes capturing skills and abilities across home, school/work and community settings, parent reports are still subject to placebo responses and/or inconsistent or inaccurate reporting for a variety of reasons. Direct observations or measurements of all adaptive skills would not be realistic in a clinical trial setting, however. The limitations of parent-report assessments as outcome measures has been discussed extensively in other reviews as well [8,9].

### 4.3. Summary and Future Directions

Overall, the results of this study indicate that, while the PEDI-CAT is more efficient and has a high concurrent validity with the VABS-3, it lacks specificity, with a poor divergent validity. Further, although our sample shared profiles of adaptive skills on the VABS-3 with those reported in previous studies, the PEDI-CAT did not yield the expected differences by gender that were identified by the VABS-3 and that are well-established as part of the FXS phenotype in other literatures [15,16,40,47,49]. Both measures showed good to excellent ICCs with repeat testing, except in the PEDI-CAT mobility domain, where it was in the moderate range, and both measures also had marked floor effects. Together, these considerations prevent us from recommending the PEDI-CAT content-balanced version at this time for Fragile X trials.

The PEDI-CAT could be useful as an efficient measure in a clinical setting to evaluate adaptive functioning profiles and obtain the T-scores and percentiles needed to establish a medical diagnosis of ID and document functioning in the disability range for services and disability supports. The PEDI-CAT does not generate a single adaptive summary score, however, that would be equivalent to the adaptive behavior composite of the VABS-3. Thus, domain scores and percentiles would need to be utilized and could lead to some challenges in diagnostic categorization if some subdomains fell above the disability range in higher-functioning patients with FXS.

When considering future directions, administering other versions of the PEDI-CAT could deepen our understanding of PEDI-CAT performances, not just for individual progress monitoring (for which the content-balanced version is suggested) but, also, for efficient measurements in lower-functioning groups (as the speedy version has been suggested) or in the newer PEDI-CAT-ASD, which has additional items adjusted for behaviors and developmental patterns in ASD. Adaptations to the PEDI-CAT may also provide an opportunity to address the issue of floor effects evidenced in both the PEDI-CAT and VABS-3 for this population. As the PEDI-CAT publishers have previously made adaptations to items tailored to specific populations, such as ASD [25,38], this could be considered for FXS as well; however, that would require additional validation.

## 5. Conclusions

Overall, the continued examination of adaptive functioning measures is important in FXS. The thoughtful catering of items or scoring methods for the FXS population may serve useful in identifying efficient and meaningful measures of adaptive functioning in FXS that can be utilized to capture changes with both short and long-term interventions for males and females across the age span.

## Figures and Tables

**Table 1:** Time 1 scores on the PEDI-CAT (Pediatric Evaluation of Disability Inventory- Computer Adaptive Test) and VABS-3 (Vineland Adaptive Behavior Scales-3rd Edition).

	Males	Females	Total	*t*-Test and Fisher’s Exact Test Results
PEDI-CAT Scores *N*	27	15	42	
Daily Activities T-score: M (SD)	21.1 (13.5)	34.0 (20.1)	25.7 (17.1)	t(40) = −2.222, *p* = 0.037 *
Range	9–47	9–64	9–64	
<5th percentile (%)	77.8%	33.3%	61.9%	
Mobility T-score: M (SD)	24.9 (16.8)	34.5 (20.8)	28.4 (18.6)	t(40) = −1.634, *p* = 0.110
Range	9–58	9–63	9–63	
<5th percentile (%)	66.7%	20.0%	50.0%	
Social-Cognitive T-score: M (SD)	17.6 (10.9)	24.5 (15.4)	20.1 (12.9)	t(40) = −1.688, *p* = 0.09
Range	9–38	9–57	9–57	
<5th percentile (%)	92.6%	66.7%	83.3%	
Responsibility T-score: M (SD)	24.9 (16.2)	34.7 (17.7)	28.4 (17.2)	t(40) = −1.818, *p* = 0.077
Range	9–60	9–59	9–60	
<5th percentile (%)	70.4%	33.3%	57.1%	
Administration Time: M (SD) (min.)	23.0 (8.8)	18.5 (8.9)	21.4 (9.5)	t(40) = 1.586, *p* = 0.121
Administration Time: Range (min.)	13–45	8–36	8–45	
VABS-3 *N*	25	14	39	
Adaptive Composite: M (SD)	47.1 (19.5)	63.6 (25.5)	52.55 (22.7)	t(31) = −2.066, *p* = 0.047 *
Range	20–80	20–87	20–87	
<5th percentile (%)	90.9%	45.5%	75.8%	
Communication: M (SD)	39.2 (20.9)	60.6 (23.7)	46.9 (24.0)	t(37) = −2.926, *p* = 0.006 **
Range	20–76	20–89	20–89	
<5th percentile (%)	92.0%	64.3%	82.1%	
Daily Living Skills: M (SD)	49.1 (22.1)	67.8 (30.4)	55.2 (26.2)	t(37) = −2.124, *p* = 0.041 *
Range	20–87	20–107	20–107	
<5th percentile (%)	88.0%	50.0%	75.7%	
Socialization: M (SD)	49.0 (20.3)	67.8 (26.0)	55.1 (23.7)	t(37) = −2.402, *p* = 0.022 *
Range	20–92	20–97	20–97	
<5th percentile (%)	92.0%	50.0%	78.4%	
Motor Skills: M (SD)	53.4 (21.0)	75.7 (81.0)	60.8 (22.7)	t(28) = −2.830, *p* = 0.009 **
Range	20–85	33–93	20–93	
<5th percentile (%)	80.0%	30.0%	78.4%	

**Table 2:** Description of participants at time 1.

	Males	Females	Total	*t*-Test and Fisher’s Exact Test Results
Age (*N*)	27	15	42	
M (SD)	13.4 (7.3)	17.6 (16.1)	14.9 (11.2)	t(40) = −1.180, *p* = 0.346
Range	4.9–31.0	1.6–50.9	1.6–50.9	
Ethnicity: Hispanic *N* (%)	4 (14.8%)	1 (6.7%)	5 (11.9%)	*p* = 0.639
Race: Caucasian *N* (%)	19 (90.5%)	12 (85.7%)	31 (88.6%)	*p* = 0.455
Stanford Binet—5 ^ *N*	25	12	37	
ABIQ—M (SD)	50.7 (7.2)	71.1 (19.9)	57.3 (15.8)	t(35) = −3.436, *p* = 0.005 **
ABIQ—Range	47–70	47–97	47–97	
FSIQ—M (SD)	45.1 (10.1)	66.9 (18.3)	52.6(16.9)	t(33) = −3.840, *p* = 0.002 **
FSIQ—Range	40–84	40–98	40–98	
FSIQz deviation score—M (SD)	42.5 (19.0)	73.5 (18.6)	53.1 (23.8)	t(33) = −4.625, *p* = 0.000 ***
FSIQz deviation score—Range	10.5–90.5	35.1–105.4	10.5–105.4	
Lifetime ASD Diagnosis *N* (%)				
No ASD	5 (18.5%)	13 (86.7%)	18 (42.9%)	*p* = 0.000 ***
ASD	22 (81.5%)	2 (13.3%)	24 (57.1%)	
Aberrant Behavior Checklist (ABC-C) *N*	17	10	27	
Total Score—M (SD)	51.8 (28.2)	26.7 (24.3)	42.5 (29.1)	t(25) = 2.348, *p* = 0.027 *
Total Score—Range	9–103	0–77	0–103	

* *p* < 0.05, ** *p* < 0.01 and *** *p* < 0.001. ABIQ = abbreviated intelligence quotient, FSIQ = full-scale intelligence quotient, NVIQ = nonverbal intelligence quotient, VIQ = verbal intelligence quotient and ASD = autism spectrum disorder. ^ Stanford Binet Intelligence Scales, Fifth Edition (SB-5) ABIQ scores available for 37 participants and SB-5 FSIQ and FSIQz scores available for 23 of 25 males and 12 of 12 females.

**Table 3:** Convergent and divergent validity: Spearman’s rho correlation coefficients.

		VABS-3 Adaptive Behavior Scales-3rd Edition Domain Standard Scores				
		Communication	Daily Living Skills	Socialization	Motor Composite (*N* = 10)	Adaptive Composite
PEDI-CAT T-scores	Daily Activities	0.769 **^,^^†^	0.664 **	0.692 **^,^^†^	0.722 **^,^^†^	0.719 **^,^^†^
	Mobility	0.551 *	0.454	0.526 *	0.463 ^‡^	0.511 *
	Social-Cognitive	0.711 **^,^^†^	0.643 **	0.653 **	0.610 *	0.676 **
	Responsibility	0.678 **^,^^†^	0.454	0.653 **	0.496	0.618 **

* Correlation is significant at the 0.05 level (2-tailed). ** Correlation is significant at the 0.01 level (2-tailed). ^†^ Correlation remained significant after control for multiple comparisons. ^‡^ Correlation approaching significance, *p* = 0.071. PEDI-CAT = Pediatric Evaluation of Disability Inventory-Computer Adaptive Test and VABS-3 = Vineland Adaptive Behavior Scales-3rd Edition.

**Table 4:** Percentage of participants earning lowest score (floor effect) on comparable domains of the VABS-3 and PEDI-CAT at time 1.

	VABS-3	PEDI-CAT	Chi-Square
VABS-3 Domain/PEDI-CAT Domain			
Daily Living Skills/Daily Activities	24.3%	38.1%	*X*^2^ (1, *N* = 79) = 1.73, *p* > 0.05
Daily Living Skills/Responsibility	24.3%	26.2%	*X*^2^ (1, *N* = 79) = 0.09, *p* > 0.05
Motor Skills/Mobility	10.0%	35.7%	*X*^2^ (1, *N* = 72) = 6.17, *p* < 0.01 **
Socialization/Social-Cognitive	16.2%	50.0%	*X*^2^ (1, *N* = 79) = 9.98, *p* < 0.01 **
Communication/Social-Cognitive	35.9%	50.0%	*X*^2^ (1, *N* = 81) = 1.64, *p* > 0.05

PEDI-CAT = Pediatric Evaluation of Disability Inventory-Computer Adaptive Test and VABS-3 = Vineland Adaptive Behavior Scales-3rd Edition.

**Table 5:** Test-retest reliability of PEDI-CAT and VABS-3: Spearman’s rho correlations and intraclass correlation coefficients (ICCs).

VABS-3 and PEDI-CAT Scores	Time 1 M (SD)	Time 2 M (SD)	Rho ^†^	ICC (95% CI) ^a^
VABS-3 *N* = 19				
Adaptive Composite Standard Score (SS)	53.6 (22.1)	55.7 (22.1)	0.769 **	0.806 (0.468–0.940)
Communication Domain SS	48.3 (25.2)	47.7 (27.5)	0.821 **	0.830 (0.527–0.945)
Daily Livings Skills Domain SS	57.6 (25.8)	60.3 (29.5)	0.833 **	0.852 (0.584–0.952)
Socialization Domain SS	56.4 (22.7)	60.2 (21.9)	0.910 **	0.882 (0.671–0.962)
Motor Skills Domain SS	59.1 (22.0)	57.9 (30.9)	0.771 **	0.817 (0.409–0.952)
PEDI-CAT Scores *N* = 19				
Daily Activities Scaled ^b^ Score	55.4 (6.7)	56.0 (5.8)	0.927 **	0.929 (0.829–0.972)
Daily Activities T-score	26.0 (15.2)	23.7 (15.2)	0.957 **	0.925 (0.810–0.971)
Mobility Scaled ^b^ Score	65.7 (3.9)	67.0 (3.8)	0.644 *	0.665 (0.318–0.855)
Mobility T-score	27.7 (15.4)	25.6 (15.7)	0.835 **	0.871 (0.702–0.948)
Social-Cognitive Scaled ^b^ Score	61.1 (5.5)	62.8 (4.6)	0.823 **	0.783 (0.465–0.915)
Social-Cognitive T-score	22.4 (12.7)	20.4 (12.9)	0.864 **	0.807 (0.572–0.920)
Responsibility Scaled ^b^ Score	46.0 (8.8)	46.0 (8.5)	0.797 **	0.921 (0.807–0.969)
Responsibility T-score	32.8 (15.6)	27.8 (15.6)	0.871 **	0.829 (0.530–0.936)

* *p* < 0.05, ** *p* < 0.01. ^†^ all remain significant after control for multiple comparisons. CI = confidence interval. ^a^ Two-way mixed effects single measurement absolute agreement model. ^b^ PEDI-CAT scaled scores are similar to a weighted raw score and are not age-dependent. PEDI-CAT = Pediatric Evaluation of Disability Inventory-Computer Adaptive Test; VABS-3 = Vineland Adaptive Behavior Scales-3rd Edition; CI = confidence interval.
